# Genetic Diversity in Oxytocin Ligands and Receptors in New World Monkeys

**DOI:** 10.1371/journal.pone.0125775

**Published:** 2015-05-04

**Authors:** Dongren Ren, Guoqing Lu, Hideaki Moriyama, Aaryn C. Mustoe, Emily B. Harrison, Jeffrey A. French

**Affiliations:** 1 Callitrichid Research Centre, Department of Psychology, University of Nebraska at Omaha, Omaha, NE 68182, United States of America; 2 Key Laboratory for Animal Biotechnology of Jiangxi Province and Ministry of Agriculture of China, Jiangxi Agricultural University, Nanchang, Jiangxi 330045, China; 3 Department of Biology, University of Nebraska at Omaha, Omaha, NE 68182, United States of America; 4 School of Biological Sciences, University of Nebraska Lincoln, Lincoln, NE 68588, United States of America; University of Florence, ITALY

## Abstract

Oxytocin (OXT) is an important neurohypophyseal hormone that influences wide spectrum of reproductive and social processes. Eutherian mammals possess a highly conserved sequence of OXT (Cys-Tyr-Ile-Gln-Asn-Cys-Pro-**Leu**-Gly). However, in this study, we sequenced the coding region for OXT in 22 species covering all New World monkeys (NWM) genera and clades, and characterize five OXT variants, including consensus mammalian Leu^8^-OXT, major variant Pro^8^-OXT, and three previously unreported variants: Ala^8^-OXT, Thr^8^-OXT, and Phe^2^-OXT. Pro^8^-OXT shows clear structural and physicochemical differences from Leu^8^-OXT. We report multiple predicted amino acid substitutions in the G protein-coupled OXT receptor (OXTR), especially in the critical N-terminus, which is crucial for OXT recognition and binding. Genera with same Pro^8^-OXT tend to cluster together on a phylogenetic tree based on OXTR sequence, and we demonstrate significant coevolution between OXT and OXTR. NWM species are characterized by high incidence of social monogamy, and we document an association between OXTR phylogeny and social monogamy. Our results demonstrate remarkable genetic diversity in the NWM OXT/OXTR system, which can provide a foundation for molecular, pharmacological, and behavioral studies of the role of OXT signaling in regulating complex social phenotypes.

## Introduction

Oxytocin (OXT) is a cyclic nonapeptide hormone synthesized primarily by neurons in hypothalamic nuclei. The OXT peptide is released from the posterior pituitary into the systemic circulation in response to a variety of stimuli such as suckling, parturition, and stressors [1]. OXT acts centrally to facilitate a wide spectrum of reproductive and social functions in mammals [1–4]. OXT is involved in the regulation of multiple facets of social relationships in mammals, including social monogamy [5–7]. It has been long-held that OXT is strongly conserved among eutherian mammals (‘consensus’ mammalian Leu^8^-OXT: Cys-Tyr-Ile-Gln-Asn-Cys-Pro-**Leu**-Gly) [1, 8]. Recently however, a novel OXT variant was identified in four species of New World monkeys (NWM), involving a substitution from leucine to proline at position eight (Pro^8^-OXT) [9]. However, it is currently unknown whether novel OXT variants are present throughout NWM (*Platyrrhini*), which consists of 17 genera distributed across *Cebidae*, *Atelidae*, and *Pitheciidae* clades. We therefore analyzed the genomic coding regions for OXT in 22 species representing each genus in *Platyrrhini*. Given that OXT actions are mediated by a specific G protein-coupled receptor [1], we also characterized the genomic regions coding for its receptor (OXTR). We then contrasted nucleotide and amino acid substitutions in OXTR, characterized the physicochemical properties of OXT and OXTR variants, and estimated coevolutionary relationships between OXT and OXTR. Additionally, given the relatively high percentage of NWM species exhibiting social monogamy (more than 50%) relative to other primate or mammalian clades [10], we statistically evaluated the association between OXT/OXTR variants and social monogamy.

## Materials and Methods

### Animals

As described in detail previously [11], a total of 22 NWM species were sampled, which covered all three clades, and at least one species per genus. The species, DNA source, sex, and institutional source of each sample are presented in S1 Table. All sequences of *OXTR* generated in our study were deposited in GenBank (accession numbers: KF701336-KF701379). Sequences for *OXT* and *OXTR* for all other primates (hominoid, Old World, and prosimian primates) were accessed from UCSC Gene Browser/NCBI/Ensembl.

### Ethics Statement

All samples were accessed from archival blood or tissue banks, or from extracted DNA samples provided by the institutions listed in S1 Table. As described in detail previously [11], all institutions are licensed and/or accredited by appropriate agencies (e.g., USDA, AZA). IACUC information is also provided in S1 Table where relevant.

### Amplification and Sequencing

Genomic DNA was extracted from whole blood or tissue samples using the DNeasy Blood and Tissue Kit (Qiagen) following manufacture’s protocol. Nested primers were used to amplify the OXTR region (S2 Table). All primers were designed based on the *OXT* and *OXTR* conserved genomic regions in several taxa including human, *Callithrix jacchus* and rhesus macaque (UCSC Genome Browser, http://genome.ucsc.edu/). All target regions in 22 species were amplified following manufacture’s protocol and then sequenced directly in two directions.

### Evolutionary Analysis

Sequences for *OXT* and *OXTR* for primates other than NWM were accessed from UCSC Gene Browser/NCBI/Ensembl. A molecular phylogenetic tree of *OXTR* was generated using the Maximum Likelihood method (1000 bootstrap), and the model with the lowest Bayesian Information Criterion score was selected (Tamura-parameter + G + I model) in MEGA 6.0 [12]. A Bayesian approach as implemented in MrBayes 3.1.2 was also used to infer phylogenetic relationships and to establish posterior probabilities for each node [13]. Markov Chain Monte Carlo simulations were run for 1,000,000 generations using a sample frequency of 10 and a burn-in of 25,000. Default setting for the prior probabilities on the model parameters (nst = 6) were used.

Assessment of coevolution between OXT and OXTR was evaluated according to previous methods [14]. Briefly, two pairwise evolutionary distance matrices were obtained in MEGA 6.0 using the genomic coding sequences of OXT (27 nucleotides) and OXTR (1170 nucleotides). A linear regression analysis was used to measure the correlation between pairwise evolutionary distances matrices between OXT and OXTR. The linear correlation coefficient was computed, and significance levels were tested. The isoelectric point (pI) and grand average of hydropathicity (GRAVY) of OXT and OXTR *N*-termini were predicted for representative species representing the five OXT ligands on the ExPASy Server [15].

We classified amino acid substitutions as conservative or radical according changes in polarity, charge, and volume categories: substitutions with a change in one or more categories were classified as radical, while substitutions with no changes in the three categories were classified as conservative [16].

### Classification of Social Monogamy

As described in detail previously [11], social monogamy in mammals refers to a long-term or sequential living arrangement between an adult male and an adult female: sharing the same territory, high rates of sociosexual behavior between pairmates, and often, but not always, biparental care. Classification of species as social monogamous was based on recent surveys [10, 17], and the classification does not imply that the species are characterized by genetic monogamy [18].

### Phylogeny-trait Association Analysis

The presence of a statistical association between OXTR-derived phylogeny and social monogamy was performed with BaTS phylogeny-trait analyses (version 1.0; monophyletic clade (MC) size statistics; 1,000 replicates) [19]. BaTS analysis is based on the null hypothesis (represented by the expected MC statistic) that no single tip bearing a given character trait (in our case, social monogamy) is more likely to share that trait with adjoining taxa than we would expect due to chance. BaTS incorporates statistical error arising from phylogenetic uncertainty and provides error intervals for hypothesis testing. A higher observed than expected MC value suggests an increased phylogeny-trait association, and a significant association (*P* < 0.05) between a particular trait value and its distribution on a phylogeny indicates a potential causative relationship.

## Result and Discussion

### Five OXT Variants in NWM

**Fig 1:**
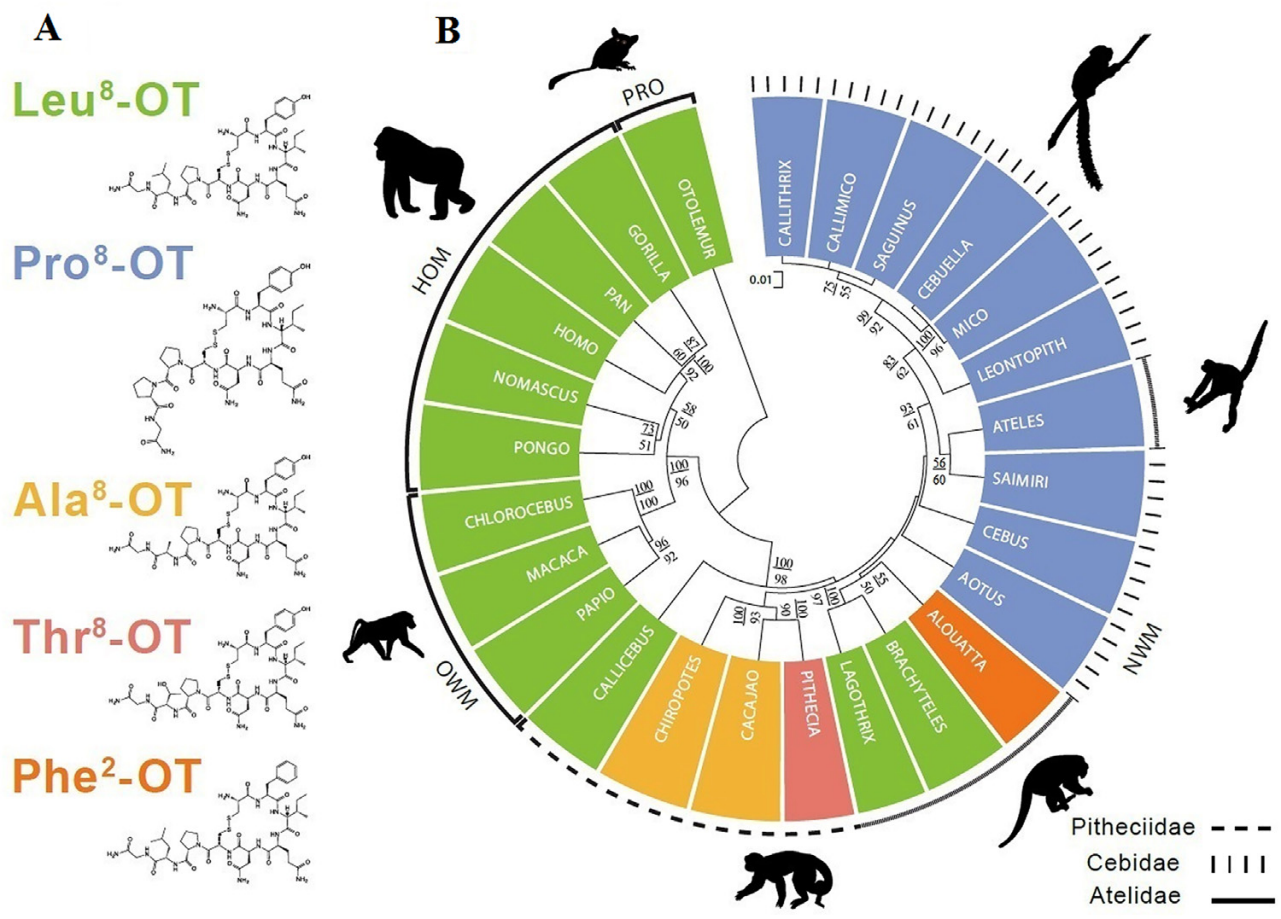
Five OXT ligands identified in New World monkeys and their distribution in a phylogenetic tree generated from OXTR nucleotide sequences. **A.** 2-D structure of mammalian consensus Leu^8^-OXT, and other four OXT variants, including Pro^8^-OXT, Ala^8^-OXT, Thr^8^-OXT and Phe^2^-OXT. The 2-D structures of oxytocin ligands were created in ChemDraw Pro 12.0. Different colors indicate different OXT ligands. **B.** OXT ligands (representing by different colors as in Fig 1A) are distributed in a phylogenetic tree inferred from OXTR nucleotides. If posterior probability (upper) and bootstrap support (lower) are < 50, no value is shown at nodes. Scale bar indicates the branch length in nucleotide substitutions per site.

We identified five distinct OXT ligands in NWM. The five ligands were ‘consensus’ mammalian OXT (Leu^8^-OXT), the most common OXT variant, Pro^8^-OXT, and three previously unreported OXT variants (Ala^8^-OXT, Thr^8^-OXT and Phe^2^-OXT; Fig 1A, S3 Table). At least one genus in all three NWM clades possessed non-consensus mammalian OXT (i.e., a ligand other than Leu^8^-OXT); thus, OXT ligand variation is widespread in NWM. The consensus phylogeny suggests that *Platyrrhini* and *Catarrhini* shared a common ancestor ~ 43.5 million years ago (MYA), with the *Platyrrhini* emerging 20–27 MYA [20]. *Pitheciidae*, the first of the three *Platyrrhini* clade emerged ~20.5 MYA and display three OXT ligands (Leu^8^, Thr^8^, and Ala^8^; the latter two variants appearing in genera that emerged ~ 14 MYA) [20–22]. *Cebidae* and *Atelidae*, sister clades to *Pitheciidae*, later emerged and radiated at about 20 MYA. All *Cebidae* express Pro^8^-OXT, and *Atelidae* display three OXT ligands (Leu^8^, Pro^8^, and Phe^2^). It is likely that the ancestor of NWM expressed Leu^8^-OXT, since this OXT ligand appears to be ancestral and is found in two of three NWM clades and in all available sequences of *Catarrhini*. Thus, considerable differentiation of the OXT coding region continued after the separation of the three NWM clades, suggesting multiple substitution events leading to OXT ligand diversity.

**Fig 2:**
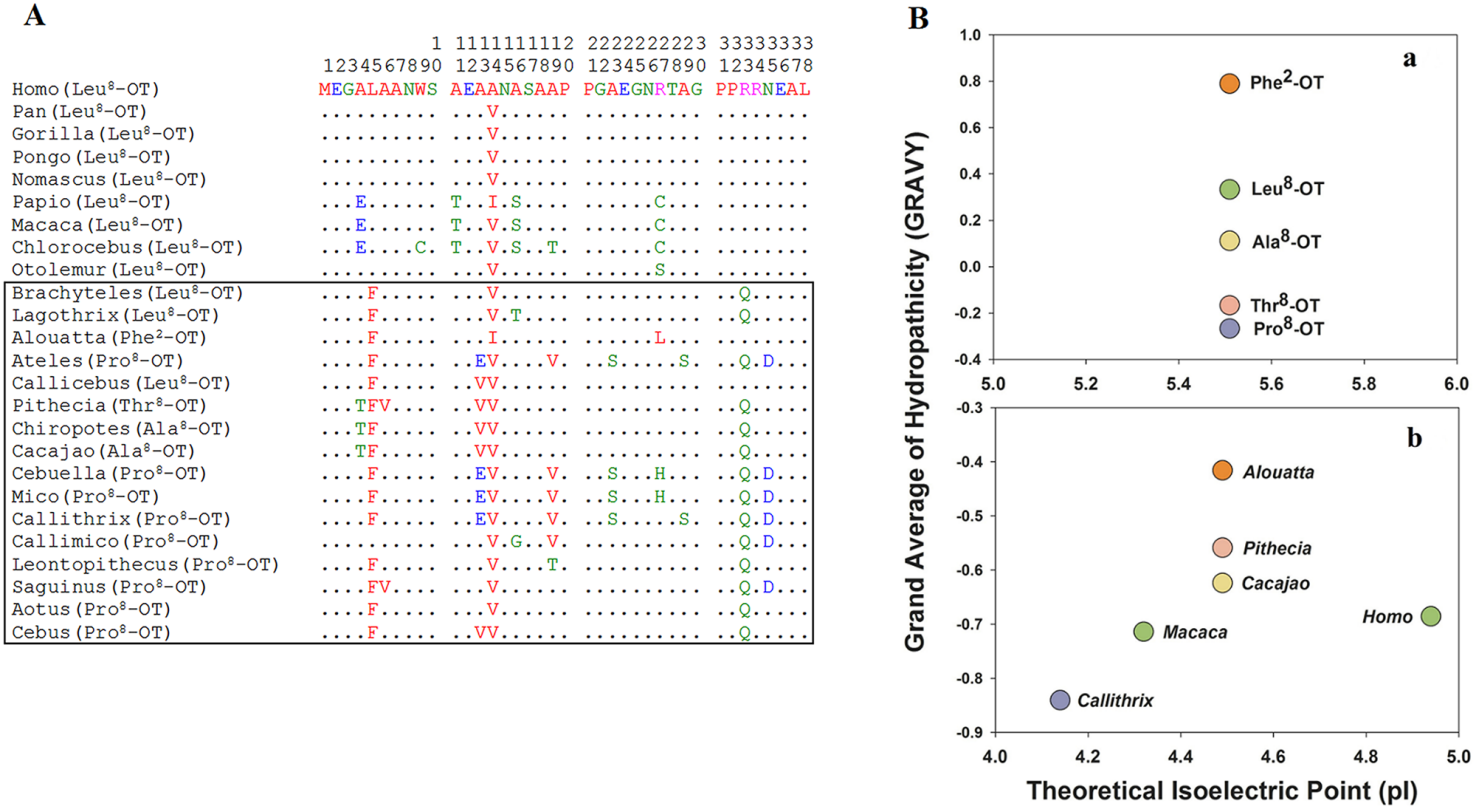
Analysis of *N*-terminus of OXTR in primates. **A.** Alignment of the 38 OXTR amino acids in New World monkeys (NWM, rectangle framed) and non-NWM primates. Sequences for non-NWM primates accessed from NCBI, UCSC or Ensembl. A dot represents identity with human OXTR amino acid. **B.** The isoelectric point (pI) and grand average of hydropathicity (GRAVY) of OXT (top) and *N*-termini of OXTR (bottom). Plots include Pro^8^-OXT species (*Callithrix*) and non-Pro^8^-OXT species (*Homo*, *Macaca*, *Alouatta*, *Pithecia* and *Cacajao*). All OXT ligands show the same pI value, but exhibit marked differences in GRAVY, with Pro^8^-OXT being most hydrophilic. *N*-termini of *Callithrix* OXTR, possessing lower pI and GRAVY values, are distinct from *N*-termini from other representative species. Symbol colors for OXTR correspond to ligand variation. Larger negative numbers in GRAVY indicate a more hydrophilic molecule; pI values less than 7.3 indicate that molecules carry a net negative charge, relative to the pH in brain tissue.

When comparing the sequences of OXT-like nonapeptides across vertebrates, positions 1, 6, 7 and 9 are generally conserved, relative to the more variable positions 2–5 and 8 [1, 23]. The neurohypophyseal nonapeptides are classified into OXT and arginine vasopressin (AVP) families based on the amino acid at position eight [1]. Compared with other OXT residues, the eighth amino acid appears to be most critical for biological functions regulated by OXT [24], and a single amino acid substitution can dramatically alter the structure, physicochemical properties, and physiological properties of OXT [8, 25]. All amino acid substitutions in NWM OXT variants have at least one physicochemical change from the corresponding amino acids in consensus mammalian OXT [16], and thus represent radical substitutions. Three variants (Ala^8^, Thr^8^, and Phe^2^) have modest changes in OXT structure relative to consensus mammalian Leu^8^-OXT (Fig 1A). Pro is the only amino acid where the side chain connects to the protein backbone twice, and Pro adds a tight turn structure that changes the direction of the polypeptide chain [26]. As a consequence, Pro^8^-OXT presents as a radically different structure from the other OXT ligands. Although all OXT ligands possess the same isoelectric point (pI), we noted marked differences in grand average of hydropathicity (GRAVY) across OXT ligands, with Pro^8^-OXT being the most hydrophilic (Fig 2B). The Pro^8^ substitution in OXT leads to an alteration in molecular structure, particularly in the linear portion of the ligand (amino acids 7–9; Fig 1A), which interacts with the OXTR *N*-terminal domain [27, 28].

### OXTR Diversity and Coevolution with OXT

**Fig 3:**
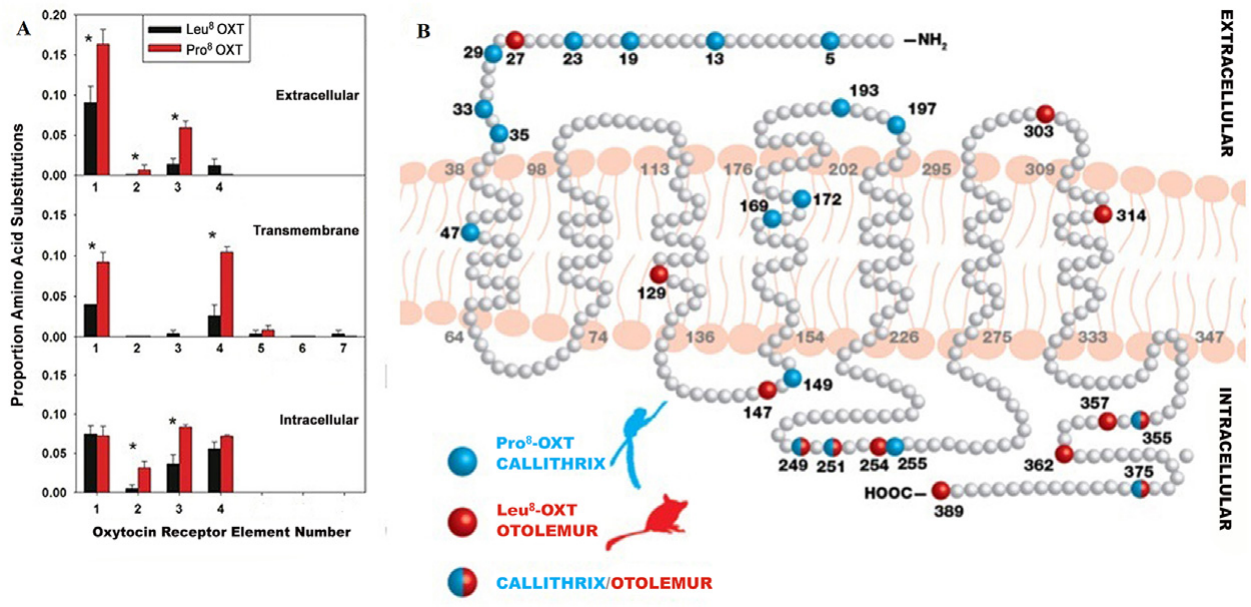
OXTR structural variation in primates with Leu^8^-OXT and Pro^8^-OXT. **A.** Proportion of amino acid substitutions across species (relative to human OXTR) in the four extra- and intracellular and seven transmembrane elements of the OXTR. Values represent mean ± SEM for primates with Leu^8^-OXT (n = 11; OXTR sequences of *Tarsius* and *Microcebus* are not available) and Pro^8^-OXT (n = 10). Sample sizes were not sufficient to include species with Ala^8^-, Thr^8^-, and Phe^2^-OXT in this analysis. Asterisks indicate significant differences (independent samples *t* test, *P* < 0.02). **B.** Representative OXTR models for species with Pro^8^-OXT (*Callithrix*) and Leu^8^-OXT (*Otolemur*), relative to human OXTR (coloured molecules represent substitutions). Although *Callithrix* is phylogenetically closer to human than *Otolemur* based on conventional phylogeny, *Callithrix* OXTR residues are more variable than *Otolemur*, especially in the *N*-terminus.

Given the diversity in OXT ligand structure in NWM, we expected corresponding changes in the sequences of NWM OXTR. Our examination of OXTR sequences, relative to the human OXTR, showed that the proportion of predicted OXTR amino acid substitutions in genera with Pro^8^-OXT was significantly higher than in genera with Leu^8^-OXT (Fig 3A). For example, although *Callithrix* (Pro^8^-OXT) is phylogenetically closer to human (Leu^8^-OXT) than the prosimian *Otolemur* (Leu^8^-OXT) [20], *Callithrix* shows more differences in OXTR sequences than *Otolemur* (Fig 3B). Since the overall three-dimensional OXTR architecture plays a significant role in ligand-receptor interactions and subsequent intracellular processes [24], it is likely there are complicated alterations in OXTRs that do not correspond in a straightforward manner to differences in OXT ligand structure.

We expected that changes in OXT ligand structure would correspond to changes in the *N*-terminus of the OXTR, which is involved in OXT recognition and binding [27, 28]. The *N*-terminus showed significantly higher proportions of amino acid substitutions (16.3%) in species with Pro^8^-OXT than in species with consensus Leu^8^-OXT (9.1%; Figs 2A and 3A). Multiple substitutions in the *N*-terminus constituted radical physicochemical changes (S4 Table). The pI and GRAVY plots for selected primate OXTR *N*-termini (Fig 2B) reveal distinct physicochemical characteristics among species with different OXT ligands, and *Callithrix* OXTR has the lowest pI and GRAVY scores. The physiochemical properties of Pro^8^-OXT and *Callithrix* OXTR suggest a high binding potential because both of them are hydrophilic. The OXTR *N*-terminus interacts with the linear *C*-terminal tripeptide region of OXT [27, 28].

Since four of five NWM OXT ligands have amino acid differences in this tripeptide region, it is likely that the modified *N*-termini in NWM OXTR arose in concert with changes in OXT sequence. To test this possibility, we statistically evaluated ligand-receptor coevolution by determining the correlation coefficient of pairwise evolutionary distances matrices among OXT and OXTR [14]. We found evidence supporting significant coevolution among OXT ligands and their corresponding OXTR across primates (*r* = 0.62, *P* < 0.001).

We generated a molecular phylogenetic tree for primates based on OXTR genomic sequences (Fig 1B). In general, the OXTR phylogeny corresponds to the conventional molecular phylogeny of primates on a clade level [20], with a clear separation of hominoid, Old World, New World, and prosimian primates. In the NWM, though the bootstrap and posterior probability support values for each node between clades are lower, the genera within same clade clustered together with high support values. Pro^8^-OXT was found in all the genera in *Cebidae*. Interestingly, the one exception to OXTR-conventional phylogeny correspondence is *Ateles*, whose OXTR clusters with the family *Cebidae* and not with congeners in the clade *Atelidae*. However, like Cebids, *Ateles* has a coding sequence for Pro^8^-OXT. Pro^8^-OXT distribution in OXTR tree partially supports the notion of ligand-receptor coevolution. Similar coevolutionary processes have been demonstrated in a host of pituitary peptide hormones and their cellular receptors, including LH, FSH, and prolactin [29, 30].

### OXTR Diversity and Social Monogamy

The diversity in OXTR/OXT structures among primates may have important implications for understanding diversity in social systems. In prosimian and Old World primates, the regular expression of social monogamy is rare or absent; in hominoid primates (apes and humans), social monogamy is noted only in gibbons, siamangs and humans [10]. By contrast, social monogamy is a relatively common occurrence in NWM, with more than 50% of species routinely displaying this social system [10]. Neuropeptide signaling in the brain mediated by OXTR may be an important mechanism for social bonding and affiliative behavior in a host of mammalian species [31]. We used BaTS analyses [19] to test for a phylogeny-corrected statistical association between OXTR diversity and social monogamy among primates. These phylogeny-trait analyses revealed that OXTR phylogeny was significantly associated with social monogamy when assessed across all primate genera (26 genera; observed MC = 5.85, expected MC = 2.05 [*P* = 0.001]) and also when limited to NWM (17 genera; observed MC = 5.81, expected MC = 2.06 [*P* = 0.004]). Our two findings showing ligand-receptor coevolution and phylogeny-trait association suggest that the OXTR/OXT system may be a factor contributing to social monogamy, a conclusion that is supported by pharmacological and neuroanatomical evidence that OXT is an important modulator of sociality in NWM [32, 33]. We recently demonstrated OXT ligand specificity in behavioral patterns associated with social monogamy in marmosets (*Callithrix*): pair-bonded marmosets treated with Pro^8^-OXT (but not Leu^8^-OXT) showed reduced social and sexual interactions with opposite-sex strangers [34]. These behavioral data are consistent with a functional role for OXT/OXTR signaling diversity in social monogamy.

In addition, we recently characterized coding region for arginine vasopressin (AVP), a nonapeptide neurohormone that is closely related to OXT that also plays an important role in modulating social behavior in mammals [11]. In stark contrast to the data we report in this study, our work on AVP documents no variation in AVP ligand structure and minimal variation in AVPR1a in NWM, including promoter RS3 microsatellites in AVPR1a [11, 35]. This suggests targeted selection for OXT variability in this primate taxon that is characterized by an exceptionally high incidence of social monogamy.

As described in detail previously [11], social monogamy is a complex social behavior. Recent hypotheses regarding the selective pressures leading to this trait include the difficulty of male defense of multiple females [10], protection from male infanticide [17], and certainty of paternity/genetic monogamy [36]. Our data demonstrate considerable variation in the genes coding for OXT ligands and receptors in NWM, a taxon characterized by a high incidence of social monogamy. Our data do not explicitly address the functional consequences of these coding regions variants for OXT and OXTR, and the biological significance of these variants requires confirmation from mRNA and protein analyses. However, there is a confirmation in one species of NWM (*Saimiri*) that coding sequence variation in ligand produces corresponding differences in OXT and OXTR mRNA and protein structure [9]. This variation may have arisen from one or more of the selective pressures favoring social monogamy, though other possibilities exist, including relaxed functional constraints on OXTR variability. Efforts that explore molecular modeling of neuropeptide ligand-receptor interactions, receptor affinity assays, and *in vivo* pharmacological and behavioral studies with altered OXT and OXTR will further characterize the functional role of OXT/OXTR system diversity in social monogamy in primates.

## Supporting Information

S1 Table Sample information for the New World monkeys in this study. * DNA = extracted and purified DNA sample provided by institution; otherwise, we extracted DNA from the source tissue indicated.
(DOCX)

S2 Table PCR primers used to amplify genomic coding regions of OXT and OXTR (Underlined primer are the nested primers). (DOCX)

S3 Table Oxytocin genomic coding sequence, predicted protein sequence and social monogamy status in primates (New World monkeys are shaded). (DOCX)

S4 Table Physicochemical change (radical or conservative) for each substitution in OXTR of New World monkeys. N-term = *N*-terminus; TM = transmembrane region; IC = intracellular region; EC = extracellular region; C-term, *C*-terminus.
(DOCX)
