# Supplementary material for: Genetic Diversity in Oxytocin Ligands and Receptors in New World Monkeys
Source: PLoS One. 2015 May 4;10(5):e0125775. doi: 10.1371/journal.pone.0125775 (PMC4418824; doi:10.1371/journal.pone.0125775)
Supplement: S1 Table — * DNA = extracted and purified DNA sample provided by institution; otherwise, we extracted DNA from the source tissue indicated. (DOCX) [file pone.0125775.s001.docx]

**Sample information for the New World monkeys in this study.**

| Scientific name | Common name | Sex | DNA source^1^ | Institution | Provider | IACUC^2^ |
| --- | --- | --- | --- | --- | --- | --- |
| *Cebuella pygmaea* | Pygmy marmoset | 1M, 1F | DNA | Omaha's Henry Doorly Zoo | Edward Louis, Jr., Rick Brenneman, Douglas L Armstrong | Zoo tissue bank |
| *Mico argentatus* | Silvery marmoset | 1M, 1F | Spleen | Bronx zoo, New York | D. McAloose, Jean A. Paré, Colleen McCann | Collected opportunistically at necropsy |
| *Callithrix geoffroyi* | White-headed marmoset | 3M, 3F | Tail | University of Nebraska at Omaha | Jeffrey French, Heather A. Jensen | 12-099-12 |
| *Callithrix kuhlii* | Wied's marmoset | 3M, 3F | Tail | University of Nebraska at Omaha | Jeffrey French, Heather A. Jensen | 12-099-12 |
| *Callithrix jacchus* | Common marmoset | 3M, 3F | Tail | University of Nebraska at Omaha | Jeffrey French, Heather A. Jensen | 12-099-12 |
| *Callithrix penicillata* | Black-tufted marmoset | 3M, 3F | Tail | University of Nebraska at Omaha | Jeffrey French, Heather A. Jensen | 12-099-12 |
| *Callimico goeldii* | Goeldi’s marmoset | 2M, 2F | Whole blood | Chicago Brookfield Zoo | Mark Warneke, Jay Petersen | Zoo tissue bank |
| *Leontopithecus rosalia* | Golden lion tamarin | 2M, 2F | Tail | University of Nebraska at Omaha | Jeffrey French, Heather A. Jensen | Tissue bank |
| *Saguinus midas* | Red-handed tamarin | 1M, 1F | Whole  blood | Southwest National Primate Research Center | Jerilyn Pecotte | 1243SM |
| *Cebus apella* | Tufted capuchin | 1M, 1F | Whole  blood | Alpha Genesis® Inc. | Melissa Ferguson | 11-007 |
| *Saimiri sciureus* | Common squirrel monkey | 1M, 1F | DNA | Omaha's Henry Doorly Zoo | Edward Louis, Jr., Rick Brenneman, Douglas L Armstrong | Zoo tissue bank |
| *Aotus azarae* | Azara's night monkey | 1M, 1F | Whole  blood | UT M. D. Anderson Cancer Center | Larry Williams, George W. Tustin, Brenda G. Webb | Tissue bank |
| *Ateles geoffroyi* | Geoffroy's spider monkey | 1M, 1F | DNA | Omaha's Henry Doorly Zoo | Edward Louis, Jr., Rick Brenneman, Douglas L Armstrong | Zoo tissue bank |
| *Ateles belzebuth* | White-bellied spider monkey | 1M, 1F | DNA | University of Texas at Austin | Anthony Di Fiore, Simone Loss | Tissue bank |
| *Brachyteles hypoxanthus* | Northern muriqui | 2M, 2F | DNA;  Whole  blood | Brachyteles Genomics Working Group | Karen B. Strier, Paulo B. Chaves, Sergio Lucena Mendes, Anthony Di Fiore, Valéria Fagundes,  Anthony Di Fiore, Simone Loss | Tissue bank |
| *Lagothrix lagotricha* | Brown woolly monkey | 2M, 1F | DNA | Omaha's Henry Doorly Zoo; | Edward Louis, Jr., Rick Brenneman, Douglas L Armstrong; | Zoo tissue bank |
|  |  |  | Liver | Louisville Zoo, Kentucky; | Elizabeth Rourk Hayden, Roy B. Burns, Steve Wing, | Approved by the Louisville Zoo Research Committee |
| *Lagothrix poeppigii* | Red woolly monkey | 2M, 1F | DNA | Louisville Zoo, Kentucky; | Elizabeth Rourk Hayden, Roy B. Burns, Steve Wing; | Approved by the Louisville Zoo Research Committee |
|  |  |  | Liver | University of Texas at Austin; | Anthony Di Fiore, Simone Loss | Tissue bank |
| *Alouatta caraya* | Black howler | 1M, 1F | DNA | Omaha's Henry Doorly Zoo | Edward Louis, Jr., Rick Brenneman, Douglas L Armstrong | Zoo tissue bank |
| *Callicebus cupreus* | Coppery titi monkey | 1M, 1F | Liver | California National Primate Research Center | Karen L. Bales, Tamara A.R. Weinstein | Approved by the UC-Davis IACUC |
| *Pithecia pithecia* | White-faced saki | 2M, 2F | DNA | Omaha's Henry Doorly Zoo; | Edward Louis, Jr., Rick Brenneman, Douglas L Armstrong; | Zoo tissue bank |
|  |  |  | Spleen | Bronx zoo, New York | D. McAloose, Jean A. Paré, Colleen McCann | Collected opportunistically at necropsy |
| *Chiropotes chiropotes* | [Red-backed bearded saki](http://en.wikipedia.org/wiki/Red-backed_Bearded_Saki) | 1M, 1F | DNA | Omaha's Henry Doorly Zoo | Edward Louis, Jr., Rick Brenneman, Douglas L Armstrong | Zoo tissue bank |
| *Cacajao calvus* | Bald uakari | 1M | DNA | University of Texas at Austin | Anthony Di Fiore, Simone Loss | Tissue bank |

^1^DNA = extracted and purified DNA sample provided by institution; otherwise, we extracted DNA from the tissue source indicated.

^2^IACUC, Institutional Animal Care and Use Committee.
