# Supplementary material for: Genetic Diversity in Oxytocin Ligands and Receptors in New World Monkeys
Source: PLoS One. 2015 May 4;10(5):e0125775. doi: 10.1371/journal.pone.0125775 (PMC4418824; doi:10.1371/journal.pone.0125775)
Supplement: S2 Table — (DOCX) [file pone.0125775.s002.docx]

PCR primers used to amplify genomic coding regions of OXT and OXTR (Underlined primer are the nested primers).

| Gene | Region | Forward primer (5'-3') | Reverse primer (5'-3') | Amplicon size (bp) |
| --- | --- | --- | --- | --- |
| OXT | Coding region | TCCCTTCCACAAAGCACCTCA | GGCGTCAAAATCTGCTCAGC | 485 |
| OXTR | Coding region | CGTTGTGCCCAGTGTTTCAGAT | CAGGTAGGTGGAGGCGAACAT | 643 |
|  |  | CCCGGTTTATTTCAGGGTAGA | GGTAGGTGGAGGCGAACATGC | 540 |
|  |  | ATGTTCGCCTCCACCTACCT | AAGCTGGGGTAGGTTAATTGC | 740 |
|  |  | ATGTTCGCCTCCACCTACCT | CAAGGGATGATGGAGCAGTGA | 710 |
|  |  | CGACATGATCGAGAGCACAGA | GGCTGAATCCCCTATCATCTT | 574 |
|  |  | GGCTGTTGCTCAGGAAATGG | CCCAAGGAGTGGAGCGATAC | 435 |
