# Supplementary material for: Genetic Diversity in Oxytocin Ligands and Receptors in New World Monkeys
Source: PLoS One. 2015 May 4;10(5):e0125775. doi: 10.1371/journal.pone.0125775 (PMC4418824; doi:10.1371/journal.pone.0125775)
Supplement: S3 Table — (DOCX) [file pone.0125775.s003.docx]

Oxytocin genomic coding sequence, predicted protein sequence and social monogamy status in primates (New World monkeys are shaded).

| **Clade** | **Species** | **Nucleotide sequence** | **Predicted**  **Protein sequence** | **Social monogamy** |
| --- | --- | --- | --- | --- |
| Hominidae | *Homo sapiens^1^* | TGC TAC ATC CAG AACTGC CCC CTG GGA | C Y I Q N C P L G | Yes |
|  | *Pan troglodytes^1^* | * * * * * * * * * * * * * * * * * * * * * * * * * * * | * * * * * * * * * | No |
|  | *Gorilla gorilla^1^* | * * * * * * * * * * * * * * * * * * * * * * * * * * * | * * * * * * * * * | No |
|  | *Pongo pygmaeus^1^* | * * * * * * * * * * * * * * * * * * * * * * * * * * * | * * * * * * * * * | No |
| Hylobatidae | *Nomascus leucogenys^1^* | * * * * * * * * * * * * * * * * * * * * * * * * * * * | * * * * * * * * * | Yes |
| Cercopithecidae | *Papio hamadryas^1^* | * * * * * * * * * * * * * * * * * * * * * * * * * * * | * * * * * * * * * | No |
|  | *Macaca mulatta^1^* | * * * * * * * * * * * * * * * * * * * * * * * * * * * | * * * * * * * * * | No |
|  | *Chlorocebus sabaeus^1^* | * * * * * * * * * * * * * * * * * * * * * * * * * * * | * * * * * * * * * | No |
| Atelidae | *Brachyteles hypoxanthus* | * * * * * * * * * * * * * * * * * * * * * * * * * * * | * * * * * * * * * | No |
|  | *Lagothrix lagotricha* | * * * * * * * * * * * * * * * * * * * * * * * * * * * | * * * * * * * * * | No |
|  | *Lagothrix poeppigii* | * * * * * * * * * * * * * * * * * * * * * * * * * * * | * * * * * * * * * | No |
|  | *Alouatta caraya* | * * * * T * * * * * * * * * * * * * * * * * * * * * * | * F * * * * * * * | No |
|  | *Ateles geoffroyi* | * * * * * * * * * * * * * * * * * * * * * * C * * * * | * * * * * * * P * | No |
|  | *Ateles belzebuth* | * * * * * * * * * * * * * * * * * * * * * * C * * * * | * * * * * * * P * | No |
| Pitheciidae | *Callicebus cupreus* | * * * * * * * * * * * * * * * * * * * * * * * * * * * | * * * * * * * * * | Yes |
|  | *Pithecia pithecia* | * * * * * * * * * * * * * * * * * * * * * AC* * * * | * * * * * * * T * | Yes |
|  | *Chiropotes chiropotes* | * * * * * * * * * * * * * * * * * * * * * GC* * * * | * * * * * * * A * | No |
|  | *Cacajao calvus* | * * * * * * * * * * * * * * * * * * * * * GC* * * * | * * * * * * * A * | No |
| Cebidae | *Cebus apella* | * * * * * * * * * * * * * * * * * * * * * * C * * * * | * * * * * * * P * | No |
|  | *Saimiri sciureus* | * * * * * * * * * * * * * * * * * * * * * * C * * * * | * * * * * * * P * | No |
|  | *Cebuella pygmaea* | * * * * * * * * * * * * * * * * * T * * * * C * * * * | * * * * * * * P * | Yes |
|  | *Mico argentatus* | * * * * * * * * * * * * * * * * * T * * * * C * * * * | * * * * * * * P * | Yes |
|  | *Callithrix geoffroyi* | * * * * * * * * * * * * * * * * * T * * * * C * * * * | * * * * * * * P * | Yes |
|  | *Callithrix kuhlii* | * * * * * * * * * * * * * * * * * T * * * * C * * * * | * * * * * * * P * | Yes |
|  | *Callithrix jacchus* | * * * * * * * * * * * * * * * * * T * * * * C * * * * | * * * * * * * P * | Yes |
|  | *Callithrix penicillata* | * * * * * * * * * * * * * * * * * T * * * * C * * * * | * * * * * * * P * | Yes |
|  | *Saguinus midas* | * * * * * * * * * * * * * * * * * T * * * * C * * * * | * * * * * * * P * | Yes |
|  | *Callimico goeldii* | * * * * * * * * * * * * * * * * * * * * * * C * * * * | * * * * * * * P * | Yes |
|  | *Leontopithecus rosalia* | * * * * * * * * * * * * * * * * * * * * * * C * * * * | * * * * * * * P * | Yes |
|  | *Aotus azarae* | * * * * * * * * * * * * * * * * * * * * * * C * * * * | * * * * * * * P * | Yes |
| Tarsiidae | *Tarsius syrichta^1^* | * * * * * * * * * * * * * * * * * * * * * T * * * * * | * * * * * * * * * | No |
| Galagidae | *Otolemur garnettii^1^* | * * * * * * * * * * * * * * * * * * * * * * * A* * G | * * * * * * * * * | No |
| Cheirogaleidae | *Microcebus murinus^1^* | * * * * * * * * * * * * * * * * * * * * * * * * * * * | * * * * * * * * * | No |

^1^Sequences accessed from NCBI, UCSC Genome Bioinformatics, or Ensembl. All others sequenced *de novo* in our laboratory.
