# Supplementary material for: Genetic Diversity in Oxytocin Ligands and Receptors in New World Monkeys
Source: PLoS One. 2015 May 4;10(5):e0125775. doi: 10.1371/journal.pone.0125775 (PMC4418824; doi:10.1371/journal.pone.0125775)
Supplement: S4 Table — N-term = N-terminus; TM = transmembrane region; IC = intracellular region; EC = extracellular region; C-term, C-terminus. (DOCX) [file pone.0125775.s004.docx]

Physicochemical change (radical or conservative) for each substitution in OXTR of New World monkeys. N-term = *N*-terminus; TM = transmembrane region; IC = intracellular region; EC = extracellular region; C-term, *C*-terminus.

| **GPCR Element** | **Position** | **Amino acid in human** | **Amino acid in NWM** | **Substitution type** |
| --- | --- | --- | --- | --- |
| *N*-term | 4 | A; polar | T; nonpolar | Radical |
|  | 5 | L; Nonpolar/relatively small | F; Nonpolar/relatively large | Radical |
|  | 6 | A; Neutral/small | V; Nonpolar/relatively small | Radical |
|  | 13 | A; Neutral, nonpolar, neutral/small | E; Negative, polar, polar/relatively small | Radical |
|  |  | A; Neutral/small | V; Nonpolar/relatively small | Radical |
|  | 14 | A; Neutral/small | V; Nonpolar/relatively small | Radical |
|  |  | A; Neutral/small | I; Nonpolar/relatively small | Radical |
|  | 16 | A; Nonpolar | G; Polar | Radical |
|  | 19 | A; Neutral/small | V; Nonpolar/relatively small | Radical |
|  |  | A; Nonpolar | T; polar | Radical |
|  | 23 | A; Nonpolar | S; Polar | Radical |
|  | 27 | R | H | Conservative |
|  |  | R; Positive, polar, polar/relatively large | L; Neutral, nonpolar, nonpolar/relatively small | Radical |
|  | 29 | A; Nonpolar | S; Polar | Radical |
|  | 33 | R; Positive, polar/relatively large | Q; Neutral, polar/relatively small | Radical |
|  | 35 | N; Neutral | D; Negative | Radical |
| TM1 | 41 | V | I | Conservative |
|  | 47 | C; Special | S; Neutral/small | Radical |
|  | 48 | L | V | Conservative |
|  | 51 | L; Nonpolar/relatively small | F; Nonpolar/relatively large | Conservative |
|  | 58 | A; Neutral/small | V; Nonpolar/relatively small | Radical |
| IC1 | 69 | Q; Neutral, polar/relatively small | H; Positive, polar/relatively large | Radical |
| TM2 |  |  |  |  |
| EC2 | 103 | Y; Polar | F; Nonpolar | Radical |
| TM3 | 135 | L | V | Conservative |
| IC2 | 141 | I | V | Conservative |
|  | 143 | Q; Neutral, polar/relatively small | K; Positive, polar/relatively large | Radical |
|  | 149 | R; Positive, polar/relatively large | S; Neutral, neutral/small | Radical |
| TM4 | 162 | L; Nonpolar/relatively small | F; Nonpolar/relatively large | Conservative |
|  | 169 | A; Neutral/small | V; Nonpolar/relatively small | Radical |
|  | 172 | V | M | Conservative |
|  | 177 | L | M | Conservative |
| EC3 | 182 | D | E | Conservative |
|  | 193 | Q; Neutral | E; Negative | Radical |
|  | 197 | P; Nonpolar | S; Polar | Radical |
| TM5 | 213 | V; Neutral, nonpolar, nonpolar/relatively small | D; Negative, polar, polar/relatively small | Radical |
|  | 214 | I; Nonpolar, nonpolar/relatively small | T; Polar, neutral/small | Radical |
|  |  | I | M | Conservative |
|  | 218 | A; Nonpolar | T; polar | Radical |
| IC3 | 236 | A; Neutral/small | V; Nonpolar/relatively small | Radical |
|  | 247 | A; Nonpolar | S; Polar | Radical |
|  | 248 | A; Neutral/small | V; Nonpolar/relatively small | Radical |
|  | 249 | A; Neutral/small | V; Nonpolar/relatively small | Radical |
|  | 251 | D; Negative, polar, polar/relatively small | A; Neutral, nonpolar, neutral/small | Radical |
|  |  | D; Negative, polar, polar/relatively small | P; Neutral, nonpolar, neutral/small | Radical |
|  |  | D; Negative, polar/relatively small | T; Neutral, neutral/small | Radical |
|  |  | D; Negative, polar, polar/relatively small | V; Neutral, nonpolar; nonpolar/relatively small | Radical |
|  | 255 | V | M | Conservative |
|  | 258 | A; Nonpolar | G; Polar | Radical |
|  | 263 | V | I | Conservative |
| TM6 |  |  |  |  |
| EC4 | 302 | A; Neutral/small | V; Nonpolar/relatively small | Radical |
| TM7 |  |  |  |  |
| *C*-term | 345 | L | M | Conservative |
|  | 349 | A; Nonpolar | S; Polar | Radical |
|  | 355 | R; Positive, polar/relatively large | N; Neutral, polar/relatively small | Radical |
|  | 357 | L; Nonpolar, nonpolar/relatively small | Q; Polar, polar/relatively small | Radical |
|  | 375 | H; Positive, polar/relatively large | Q; Neutral, polar/relatively small | Radical |
|  | 388 | T; Polar, neutral/small | M; Nonpolar, nonpolar/relatively small | Radical |
